# Augmented Pain Processing in Primary and Secondary Somatosensory Cortex in Fibromyalgia: A Magnetoencephalography Study Using Intra-Epidermal Electrical Stimulation

**DOI:** 10.1371/journal.pone.0151776

**Published:** 2016-03-18

**Authors:** Manyoel Lim, Meyke Roosink, June Sic Kim, Hye Won Kim, Eun Bong Lee, Kyeong Min Son, Hyun Ah Kim, Chun Kee Chung

**Affiliations:** 1 Neuroscience Research Institute, Seoul National University College of Medicine, Seoul, Korea; 2 Department of Rehabilitation, Radboud University Medical Center, Nijmegen, the Netherlands; 3 Department of Brain and Cognitive Sciences, Seoul National University College of Natural Sciences, Seoul, Korea; 4 Division of Rheumatology, Department of Internal Medicine, Seoul National University College of Medicine, Seoul, Korea; 5 Division of Rheumatology, Department of Internal Medicine, Eulji University School of Medicine, Eulji General Hospital, Seoul, Korea; 6 Department of Internal Medicine, Hallym University College of Medicine, Chuncheon, Korea; 7 Division of Rheumatology, Department of Internal Medicine, Hallym University Sacred Heart Hospital, Anyang, Korea; 8 Department of Neurosurgery, Seoul National University Hospital, Seoul, Korea; University Medical Center Goettingen, GERMANY

## Abstract

The aim of this study was to investigate augmented pain processing in the cortical somatosensory system in patients with fibromyalgia (FM). Cortical evoked responses were recorded in FM (n = 19) and healthy subjects (n = 21) using magnetoencephalography after noxious intra-epidermal electrical stimulation (IES) of the hand dorsum (pain rating 6 on a numeric rating scale, perceptually-equivalent). In addition, healthy subjects were stimulated using the amplitude corresponding to the average stimulus intensity rated 6 in patients with FM (intensity-equivalent). Quantitative sensory testing was performed on the hand dorsum or thenar muscle (neutral site) and over the trapezius muscle (tender point), using IES (thresholds, ratings, temporal summation of pain, stimulus-response curve) and mechanical stimuli (threshold, ratings). Increased amplitude of cortical responses was found in patients with FM as compared to healthy subjects. These included the contralateral primary (S1) and bilateral secondary somatosensory cortices (S2) in response to intensity-equivalent stimuli and the contralateral S1 and S2 in response to perceptually-equivalent stimuli. The amplitude of the contralateral S2 response in patients with FM was positively correlated with average pain intensity over the last week. Quantitative sensory testing results showed that patients with FM were more sensitive to painful IES as well as to mechanical stimulation, regardless of whether the stimulation site was the hand or the trapezius muscle. Interestingly, the slope of the stimulus-response relationship as well as temporal summation of pain in response to IES was not different between groups. Together, these results suggest that the observed pain augmentation in response to IES in patients with FM could be due to sensitization or disinhibition of the cortical somatosensory system. Since the S2 has been shown to play a role in higher-order functions, further studies are needed to clarify the role of augmented S2 response in clinical characteristics of FM.

## Introduction

Fibromyalgia (FM) is commonly associated with chronic wide-spread pain, but is notorious for its multi-modal symptoms such as anxiety and depression, sleep disturbances, fatigue and cognitive symptoms [1]. Typically, patients report pain upon palpation of pre-defined tender point sites, and the number of painful tender points (at least 11 out of 18) has been commonly used as a diagnostic criterion for FM (American College of Rheumatology, 1990) [2].

Previous studies of FM have shown that pain sensitivity [3–7], temporal summation of pain [8–11], and cortical evoked responses [12–18] are generally increased in patients with FM as compared to healthy control (HC) subjects, and that patients with FM often report longer and different after-sensations [3, 8]. Moreover, patients with FM may show reduced endogenous pain inhibitory functions [5, 6, 19–21] and abnormal responses to innocuous somatosensory [5, 22–24], auditory [25–27], and olfactory stimuli [28]. Together, these findings suggest that sensory augmentation could be “generalized”, i.e. occurring at the central level and not being specific for pain, although some inconsistencies remain [4, 29–31].

What remains largely unclear from studies assessing sensory symptoms and signs in patients with FM is how different outcome measures relate to each other. This is particularly important since patients with FM are notorious for their heterogeneity, and symptoms tend to evolve over time. Also, the pathophysiology of FM, and in particular the relative contributions of different pain mechanisms (e.g. increased facilitation and/or decreased inhibition) at different neural levels (i.e. peripheral afferent, spinal cord and/or brain), remains a matter of debate [1, 28, 32–34]. A better understanding of the mechanisms underlying pain augmentation may help to develop pathophysiological models of FM and may contribute to improved diagnosis and treatment.

With respect to the stimulation method, a recent magnetoencephalography (MEG) study used painful and non-painful mechanical pressure stimuli that were subjectively matched between patients with FM and HC subjects [17]. Since painful mechanical pressure stimulation typically activates Aβ-, Aδ- and C-fibers, it is necessary to subtract the Aβ-fiber mediated response to assess the pain-related brain response, as in previous functional magnetic resonance imaging (MRI) studies in FM [13]. Otherwise, both intra-epidermal electrical stimulation (IES) [35–40] and laser stimulation [41–44] have been regarded as validated methods for selective activation of Aδ nociceptors in the epidermis. Prior MEG studies in combination with IES [40, 45, 46] or laser stimulation [42–44, 47, 48] have reported activation of several brain areas involved in the processing of the sensory-discriminative component of pain, such as the primary (S1) and secondary (S2) somatosensory cortices and/or posterior parietal cortex (PPC). Also recently, Mouraux et al. (2014) showed that trains of repeated IES with a short inter-stimulus interval can be used to increase the intensity of perception and brain responses [38].

The present study aimed to investigate the central augmentation of pain processing in patients FM using MEG and IES which can selectively stimulate cutaneous Aδ nociceptors [35]. Quantitative sensory testing was performed on the hand dorsum or thenar muscle (neutral site) and over the trapezius muscle (tender point), using IES (thresholds, ratings, temporal summation of pain) and mechanical stimuli (threshold, ratings). In addition, the nature of pain augmentation (e.g. higher offset and/or increased slope) was assessed using a stimulus-response curve for standardized IES on the hand dorsum. Cortical pain processing of noxious stimuli was prioritized for the estimation in the S1 and S2 [46].

## Materials and Methods

### Subjects

Subjects were right-handed (as confirmed by the Edinburgh Handedness Inventory) [49], females and aged between 30 and 60 years (during which patients were more likely to be diagnosed as having primary FM). Subjects were excluded from participation if they presented with contraindications for MEG and/or MRI assessment, pregnancy, a medical history of a psychiatric disease or a disorder affecting the central nervous system (i.e. cerebrovascular accident, multiple sclerosis, Parkinson’s disease), or signs of peripheral neuropathy of the upper extremities (e.g. related to trauma, polyneuropathy). Additional inclusion and exclusion criteria were specified for patients and HC subjects separately as mentioned below.

#### Patients

Patients were recruited from the outpatient clinics of the rheumatology departments of the Seoul National University Hospital and Hallym University Sacred Heart Hospital. Patients were eligible for participation if they were clinically diagnosed as having primary FM (according to the American College of Rheumatology 1990 criteria [2] that are still commonly used for research purposes [21, 23, 50, 51]), their disease duration was at least 3 months, they reported an average pain intensity of at least 40 on a 0−100 mm visual analogue scale (VAS; 0 mm = no pain, 100 mm = worst pain imaginable) during the past week, and they were willing to stop taking medications known to influence the somatosensory system (e.g. analgesics, antidepressants, anticonvulsants) at least 3 days prior to assessments. In addition to the general exclusion criteria, patient-specific exclusion criteria were secondary FM, concomitant chronic pain of other etiology (i.e. rheumatoid arthritis, osteoarthritis), concomitant acute pain in the upper extremities (e.g. due to injury).

#### Healthy controls

HC subjects were recruited through local advertisements and were matched for age and gender to the FM group. In addition to the general exclusion criteria, control-specific exclusion criteria were chronic pain complaints of any kind, and acute pain at the time of the assessments.

#### Ethics

The study protocol was approved by the institutional review board at Seoul National University Hospital (H-1107-013-367) and Hallym University Sacred Heart Hospital (2011-I048) and the study was conducted in compliance with the Declaration of Helsinki. All subjects received verbal information about the study protocol and signed the informed consent form prior to participation.

### Demographics and medical data

For all subjects, age (years), marital status (single, married, separated/divorced), education (years), employment status (employed, unemployed/housewife), and medication use were registered. For patients with FM, the disease duration (since onset of wide-spread pain) and number of tender points (as assessed by the treating rheumatologist using manual palpation upon inclusion) were registered. The tender point count was (re-)assessed by a trained rheumatologist from Seoul National University Hospital (H.W.K) in both patients and HC subjects using a hand-held pressure algometer (Baseline Evaluation Instruments, Fabrication Enterprises, New York, USA). In addition, subjects filled out the Fibromyalgia Impact Questionnaire (FIQ, patients only) [52], Beck’s Anxiety Inventory (BAI) [53], Beck’s Depression Inventory (BDI) [54], and Pittsburgh Sleep Quality Index (PSQI) [55].

The intensity of average pain over the last week was assessed using a VAS. The qualitative aspects of clinical pain perception were assessed using the short-form McGill pain questionnaire (SF-MPQ) [56].

### Quantitative sensory testing

All tests were performed on the right (dominant) upper extremity (hand and trapezius muscle). The test order was fixed. All tests were performed before MEG recordings.

#### Mechanical stimuli

The tactile detection threshold (TDT) was determined using 5 Semmes Weinstein monofilaments (sizes: 2.83, 3.61, 4.31, 4.56, 6.65; Baseline Evaluation Instruments, Fabrication Enterprises, New York, USA). The filaments were applied at 2 locations over the hand dorsum and at 2 locations over the trapezius muscle. For each body region, the TDT was defined as the smallest filament that could be perceived at each location. In addition, subjects rated the perceived painfulness of a standardized pinprick stimulus (PIN, 6.65 Semmes-Weinstein filament).

The pressure pain threshold (PPT) was determined using an experimenter-operated pressure algometer (Baseline Evaluation Instruments, Fabrication Enterprises, New York, USA). An in-house Teflon stimulation surface of 1 cm^2^ and a slope of 0.5 kg per second were used. Subjects were instructed to keep their arm in zero degrees of shoulder abduction and 90 degrees of elbow flexion. The PPT was then determined at 3 locations over the thenar eminence of the hand and at 3 locations over the trapezius muscle [4, 57, 58]. In response to the increasing pressure delivered by the experimenter, subjects were instructed to verbally indicate when they first perceived the pressure as painful. The 3 PPTs for each body region were averaged for further analysis.

For all mechanical thresholds, subjects rated the average perceived painfulness directly after threshold determination using a numeric rating scale (NRS; 0 = no pain, 10 = worst pain imaginable). All mechanical stimuli were delivered by a trained experimenter.

#### Electrical stimuli

The electrical sensory thresholds were determined using an experimenter-operated Digitimer DS7AH, which is a high voltage constant current stimulator (Digitimer Ltd., United Kingdom), and an intra-epidermal electrode (Nihon-Kohden, Tokyo, Japan) (pulse width: 1 ms) [36, 39]. The intra-epidermal electrode was placed over the skin between the first and second meta-carpal bones of the hand dorsum (first assessment) or over the skin overlying the trapezius muscle (second assessment). The electrical sensation threshold (EST) was determined by manually increasing the stimulator current output in steps of 0.05 mA. The subject had to indicate verbally when she first perceived the stimulus (typically a faint sensation). The electrical pain threshold (EPT) was determined by manually increasing the stimulator current output in steps of 0.3 mA. The subject had to verbally indicate when she first perceived the stimulus as being sharp and painful. The electrical pain tolerance threshold (EPTT) was determined by manually increasing the stimulator current output in steps of 0.5 mA. The subject had to verbally indicate when she first perceived the stimulus as being very sharp and painful, and was not willing to tolerate a higher stimulus amplitude. Prior to the actual assessments, subjects were trained to determine these thresholds reliably at both locations. All thresholds were determined 3 times for each body region. After each third assessment, subjects rated the average perceived painfulness using an NRS. Thresholds were averaged for each body region separately for further analysis.

#### Stimulus-response curve

A series of 12 standardized IES were presented to each subject on the hand dorsum. Stimulus intensities were 25%, 100%, 175% and 250% of the individual EPT, and were presented in a semi-randomized order, so that all intensities were used 3 times. The inter-stimulus interval was approximately 5–10 seconds. Subjects were asked to rate the pain intensity of each stimulus using an NRS. Ratings were averaged according to stimulus intensity for further analysis.

#### Temporal summation of pain

Lastly, subjects were asked to rate the pain intensity of a single IES and the pain intensity of the last IES in a 1 Hz pulse train of 5 pulses using an NRS. Pulse trains were generated using Stim2 software (Compumedics Neuroscan, Charlotte, North Carolina, USA). The stimulus intensity equaled the individual EPT. Each series of pulses (single, train) was rated 3 times, and then averaged. Temporal summation of pain could not be determined in 1 patient with FM due to technical problems.

### MEG responses to IES

#### Experimental conditions

An intra-epidermal electrode connected to a Digitimer DS7AH (Digitimer Ltd., United Kingdom) operated by Stim2 Software (Compumedics Neuroscan, Charlotte, North Carolina, USA), was used to apply single pulse IES to the dorsum of the right hand [35]. A pulse width of 1 ms was used.

For patients, the stimulus intensity (mA) corresponded to moderately painful stimulation with a perceptual pain rating of 6 on the NRS. This was determined after assessing electrical quantitative sensory testing (QST) on the hand, by manually increasing the stimulator current output in steps of 0.3 mA. For HC subjects, a perceptually-equivalent (HC-PE) stimulus and an intensity-equivalent (HC-IE) stimulus were used. For HC-PE, the stimulus intensity corresponded to moderately painful stimulation with a perceptual pain rating of 6 on the NRS, and it was determined for each subject individually as described for patients with FM. For HC-IE, the stimulus intensity was fixed at 2.66 mA, corresponding to the stimulus intensity necessary to evoke an average pain rating of 6 on the NRS in patients with FM (n = 7). The order of the stimulus condition (HC-IE or HC-PE) was randomized across subjects.

For each stimulus condition, a total number of 100 trials were recorded. The inter-stimulus interval was 4–6 seconds (randomized). Subjects were instructed not to pay attention to the stimuli and to fix their gaze on a cross in the center of the front wall.

#### MEG recordings

The MEG signals were recorded in a magnetically shielded room using a helmet-shaped whole-head neuromagnetometer with a sensor array of 102 identical triple sensors (VectorViewTM, Elektra Neuromag Oy, Helsinki, Finland). Each sensor consisted of 2 orthogonal planar gradiometers and 1 magnetometer. Subjects were seated comfortably under the helmet-shaped sensor array. The exact location of the head relative to the sensor array was determined by placing 4 head position indicator coils at known sites on the scalp, and the magnetic signal produced by electrical currents delivered to each of these 4 head position indicator coils was subsequently measured. The location of the head position indicator coils relative to 3 anatomical landmarks, the nasion and the 2 preauricular points, was measured using a 3-dimensional digitizer (FASTRAKTM, Polhemus, Colchester, Vermont, USA). In this way, MEG and MRI coordinate systems could be aligned [59]. The x-axis passed through the 2 preauricular points (right = positive), the y-axis (positive) passed through the nasion, and the z-axis (positive) pointed upward. High-resolution T1-weighted MR images were obtained from all subjects, except for one control subject due to claustrophobia, using a Siemens 3 T scanner (Siemens Magnetom TrioTim, Siemens, Erlangen, Germany).

The MEG signals were recorded with a band-pass filter of 0.1–300 Hz and were digitized at 1 kHz. Environmental and biological noises were reduced by applying the spatiotemporal space separation method to the acquired MEG signals using MaxFilter software version 2.2.10 (Elekta Neuromag Oy, Helsinki, Finland) [60].

#### MEG analysis

Epochs with amplitudes exceeding 3000 fT/cm (MEG) or 150 μV (electrooculogram) during the time window for analysis (100 ms pre-stimulus to 400 ms post-stimulus) were excluded from the analysis. All remaining epochs were averaged according to the stimulation intensity level. A minimum of 80 epochs were needed for subjects to be included in the analyses.

Source modeling was based on the signals recorded by the 204 gradiometers in individual subjects in response to NRS6 (patients) and HC-PE (HC subjects) stimulation. The cortical sources were modelled in the time domain as equivalent current dipoles (ECD), using Elekta Neuromag software (Elekta Neuromag Oy, Helsinki, Finland) [59]. A spherical volume conductor was used to model the head. For the subject in whom brain MRI was not available, a sphere model was fitted to accurately digitized isotrak points using MaxFilter software [61]. The ECDs that best explained the measured data were determined by a least-squares search at the latency of the peak amplitude, using a subset of 18–24 gradiometer channels over the maximum response area. This resulted in the respective three-dimensional location, orientation and strength of the ECDs in a spherical volume conductor model of the head. Only ECDs with a goodness-of-fit ≥ 80% of the field variance were accepted for further analysis [47, 62].

A source corresponding to the contralateral S1 (cS1) was found in 14 FM and 15 HC subjects. Sources corresponding to the contralateral secondary (cS2) and ipsilateral secondary (iS2) somatosensory cortex were found in all but one FM subject. A source corresponding to the contralateral PPC was found in 2 FM and 2 HC subjects. For the subject in whom brain MRI was not available, sources were identified by considering coordinates, peak latencies and orientations of the modelled sources. After identifying the single dipoles for each subject, the entire time window for analysis and all channels were taken into account for computing an individual time-varying multi-dipole model with fixed ECDs. For those subjects in whom a PPC source was identified, this source was included in the multi-dipole model to improve its goodness-of-fit. However, no further analysis relating to PPC activation was performed since only 2 PPC data sets would be available per group. For HC subjects, the sources estimated based on the HC-PE condition were applied to the signals recorded for the HC-IE condition [63]. For each subject and stimulation intensity level, the response peak latencies and amplitudes of the ECDs corresponding to the cS1, cS2 and iS2 sources were determined and used for further analysis.

Individual MRIs were spatially normalized to the Talairach coordinates using BrainVoyager QX software (version 1.10; Brain Innovation, Maastricht, The Netherlands) according to Lim et al. 2011 and 2015 [23, 64]. Locations of the cS1, cS2 and iS2 sources in the head coordinates were transformed into Talairach coordinates using Brain Electrical Source Analysis software (version 5.1.8; MEGIS software, Munich, Germany). Twenty HC subjects whose MRI data were available were included in deriving the source location in the Talairach coordinates.

### Statistical analysis

All statistical testing was performed using SPSS 13.0 for Windows (SPSS Inc. Chicago, IL, USA).

#### Demographic and medical data

For all demographic and medical data and pain assessments, frequencies (binary data) or averages and standard deviations (continuous data) were calculated for each group, and compared using appropriate statistical tests (independent t-tests and X^2^-tests). All tests were 2-tailed. A p-value less than 0.05 was considered statistically significant.

#### Quantitative sensory testing

QST parameters (thresholds, ratings, stimulus-response curve, temporal summation of pain) were generally not normally distributed. As such, median and range were calculated for thresholds and threshold ratings. For presentation purposes, the stimulus-response curves and results for temporal summation of pain are presented using mean ± SEM. Prior to statistical analyses, all individual raw QST parameters were log-transformed to allow for testing under normality assumptions [58].

For QST thresholds and ratings (TDT, PIN, PPT, EST, EPT, EPTT), differences between groups were statistically tested using repeated measures analyses of variance (ANOVA) with factors [Site] (hand, trapezius muscle) and [Group] (FM, HC). For the stimulus-response curve, ratings for the series of fixed stimulus amplitudes at 25%, 100%, 175% and 250% of the EPT were compared between groups using a repeated measures ANOVA with factors [Intensity] (25%, 50%, 100%, 175%) and [Group] (FM, HC). For temporal summation of pain, the ratings for the single pulse and pulse train stimulation at 100% of the EPT, were compared between groups using a repeated measures analysis with factors [Number of pulses] (1, 5) and [Group] (FM, HC). Differential effects of temporal summation of pain were tested as interactions between the number of pulses and group as in a previous study [10]. The influence of potential confounders was assessed using additional multivariate analyses with dependent [QST threshold and/or rating] (TDT, PIN, PPT, EST, EPT, EPTT), factor [Group] (FM, HC) and covariate [potential confounder] (BAI, BDI, PSQI). All factors were entered into the analyses separately. Although the covariates BDI (PPT) and PSQI (TDT, EST) were significant upon multivariate testing, associations for independent testing were not consistent. Moreover, in all cases, a significant effect of [Group] remained. We therefore considered systematic confounding by any of these variables highly unlikely.

#### MEG data

The peak latencies and amplitudes of the bilateral S2 sources were analysed using repeated measures ANOVA with a within-subjects factor [Source] (cS2, iS2) and between-subjects factor [Group] (FM, HC-IE or HC-PE). Mauchly's test of sphericity was used to evaluate the assumption of sphericity. Significant between-subjects effects were further tested using independent t-tests. The between-group differences in peak latencies and amplitudes of the cS1 source were compared by independent t-tests with factor [Group] (FM, HC-IE or HC-PE). Talairach coordinates of the cS1, cS2 and iS2 sources were compared separately by means of repeated measures ANOVA with coordinate (x, y and z) as a within-subject factor and group (FM and HC) as a between-subject factor. The relationship between the amplitude of cortical responses and FM pain intensity as reported for the last week (VAS) was assessed using Pearson’s correlation coefficient (r). The influence of potential confounders on bilateral S2 source amplitudes was assessed using a multivariate analysis with a dependent variables [cS2, iS2], factor [Group] (FM, HC-IE or HC-PE) and covariate [potential confounder] (BAI, BDI and PSQI). All factors were entered into the analyses separately. There were no significant effects for any of the covariates. The influence of potential confounders on cS1 source amplitudes was assessed using a multivariate analysis with dependent variable [cS1], factor [Group] (FM, HC-IE or HC-PE) and covariate [potential confounder] (BAI, BDI and PSQI). All factors were entered into the analyses separately. Although the covariate BAI was significant, the significant effect of [Group] remained. In summary, it is unlikely that the amplitudes of cortical sources were confounded by group differences in clinical variables relating to anxiety, depression or sleep quality.

## Results

A total of 19 patients with FM and 21 HC subjects participated in the experiments.

### Demographics & medical data

A summary of demographics and medical data is presented in Table 1. Patients with FM showed higher anxiety scores (BAI), higher depression scores (BDI), reduced sleep quality (PSQI) (p < 0.001), and an increased number of painful tender points upon manual palpation or pressure algometry (p < 0.001).

**Table 1:** Demographics and clinical characteristics of the participants.

	FM (n = 19)	HC (n = 21)	p [Group]
Age (years)	44.9 ± 8.3	44.8 ± 8.2	0.958
Education (years)	13.1 ± 2.2	12.9 ± 2.7	0.853
Occupation (working), n (%)	10 (53%)	13 (62%)	0.554
Marital status (married), n (%)	16 (84%)	17 (81%)	0.787
Medication, n (%)			
Analgesics/muscle relaxants/NSAIDs	14 (74%)	−	−
Antidepressants	14 (74%)	−	−
Anticonvulsants	7 (37%)	−	−
BAI (0–63)	23.3 ± 10.8	1.8 ± 2.0	**<0.001**
BDI (0–63)	19.0 ± 6.8	2.8 ± 3.9	**<0.001**
PSQI (0–21)	13.0 ± 3.4	3.1 ± 1.2	**<0.001**
TP manual (0–18)	15.7 ± 1.8	1.7 ± 2.3	**<0.001**
TP algometer (0–18)	14.2 ± 3.8	1.9 ± 1.7	**<0.001**
FIQ (0–100)	62.5 ± 13.2	−	−
Pain duration (months)	35.6 ± 31.1	−	−
Pain intensity—last week (mm)	57.2 ± 20.1	−	−
SF-MPQ sensory (0–33)	14.7 ± 6.8	−	−
SF-MPQ affective (0–12)	5.8 ± 2.6	−	−
SF-MPQ total (0–45)	20.5 ± 8.8	−	−

Data are presented as mean ± SD or number of subjects (%). Pain intensity (visual analog scale) was determined before QST and MEG assessments. FM: fibromyalgia, HC: healthy controls, NSAIDs: nonsteroidal anti-inflammatory drugs, BAI: Beck’s anxiety inventory, BDI: Beck’s depression inventory, PSQI: Pittsburgh sleep quality index, TP: tender points, FIQ: fibromyalgia impact questionnaire, SF-MPQ: short-form McGill pain questionnaire. p-values are based on independent t-tests (continuous variables) and X^2^-tests (categorical variables) with factor [Group]. Significant p-values (p < 0.05) are depicted in bold type.

### Quantitative sensory testing

#### Thresholds and ratings

A summary of the median and range of thresholds and ratings as obtained with QST is presented in Table 2. For all subjects, TDT thresholds were found to be within the normal range (Semmes Weinstein filament sizes 3.61 or 4.31) and were not analysed further. Statistical analyses revealed significant differences between groups, indicating that QST thresholds (PPT, EPT, EPTT) were lower and QST ratings (PIN, PPT, EST, EPT, EPTT) were higher in patients with FM as compared to HC subjects. With respect to the 2 stimulation sites, the PPT was found to be significantly lower (p < 0.001) and the PIN rating was significantly higher (p = 0.003) for stimulation of the trapezius muscle. No significant interaction effects were found (p > 0.05), suggesting that the effect of [Site] was similar in both groups.

**Table 2:** Quantitative sensory testing.

Test	Site	Outcome	FM (n = 19)	HC (n = 21)	p [Group]	p [Site]
TDT	H	Rating (0–10)	0 [0–0]	0 [0–0]	0.134	0.134
	T	Rating (0–10)	0 [0–1]	0 [0–0]		
PIN	H	Rating (0–10)	2 [0–5]	0 [0–3]	**<0.001**	**0.003**
	T	Rating (0–10)	3 [0–7]	0 [0–3]		
PPT	H	Threshold (kPa)	2.83 [1.53–4.73]	3.77 [2.70–4.50]	**<0.001**	**<0.001**
		Rating (0–10)	3 [1–6]	2 [1–4]	**0.005**	0.427
	T	Threshold (kPa)	2.10 [1.43–5.75]	3.17 [2.20–4.67]		
		Rating (0–10)	3 [1–7]	2 [0–4]		
EST	H	Threshold (mA)	0.15 [0.10–0.27]	0.15 [0.10–0.28]	0.856	0.388
		Rating (0–10)	0 [0–3]	0 [0–0]	**0.004**	0.131
	T	Threshold (mA)	0.15 [0.05–0.48]	0.15 [0.10–0.45]		
		Rating (0–10)	0 [0–1]	0 [0–0]		
EPT	H	Threshold (mA)	1.00 [0.60–1.80]	1.30 [0.80–3.90]	**0.004**	0.097
		Rating (0–10)	3 [1–5]	2 [1–4]	**0.001**	0.797
	T	Threshold (mA)	1.10 [0.60–2.80]	1.20 [0.80–3.50]		
		Rating (0–10)	3 [1–4]	1 [1–3]		
EPTT*	H	Threshold (mA)	2.83 [1.83–6.00]	5.33 [2.50–8.50]	**<0.001**	0.986
		Rating (0–10)	6 [4–8]	4 [2–7]	**0.003**	0.320
	T	Threshold (mA)	2.50 [1.50–7.07]	5.08 [2.33–12.50]		
		Rating (0–10)	6 [3–8]	5 [3–7]		

Data are presented as median [min—max]. FM: fibromyalgia, HC: healthy controls, TDT: tactile detection threshold, PIN: pinprick stimulation, PPT: pressure pain threshold, EST: electrical sensation threshold, EPT: electrical pain threshold, EPTT: electrical pain tolerance threshold, H: hand (dorsum or thenar muscle), T: trapezius muscle.

*Some subjects did not report tolerance at the highest stimulation level. Since this occurred in both patients and controls alike, and since we did not set a cut-off threshold (mA) for the forehand, it was decided to exclude these subjects from the EPTT analyses. As such, the final number of subjects for the EPTT analyses were: for the hand FM (n = 18), and for the trapezius muscle FM (n = 16) and HC (n = 16), and repeated measures ANOVA for the EPTT was based on FM (n = 16) and HC (n = 16). For QST thresholds and ratings, p-values were based on univariate results of repeated measures ANOVA using log-normalized data with factors [Group] and [Site]. Significant p-values (p < 0.05) are depicted in bold type. No significant interaction effects were found.

#### Stimulus-response curve

**Fig 1:**
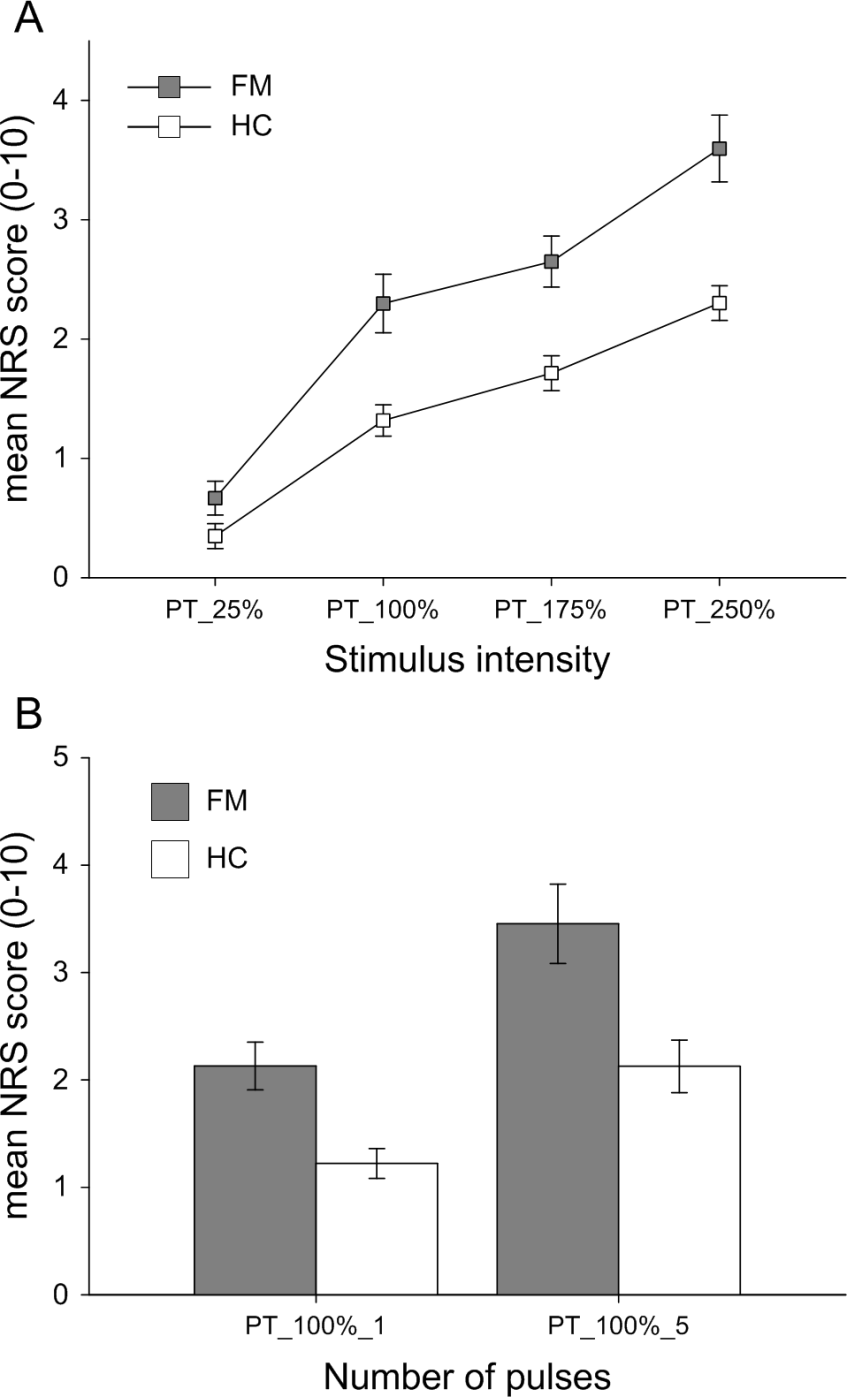
**Stimulus-response curve (A) and temporal summation of pain (B) in response to standardized intra-epidermal electrical stimulation.** Grey squares/bars represent patients with FM (A: n = 19, B: n = 18), white squares/bars represent HC subjects (n = 21). The levels of stimulation (x-axis) were based on individual pain thresholds. PT_25%, PT_100%, PT_175% and PT_250% correspond to 25%, 100%, 175% and 250% of the pain threshold intensity (mA), respectively. Data are expressed as the mean ± SEM.

The mean NRS scores corresponding to fixed levels of IES are presented for both groups in Fig 1A. Statistical analysis showed that, compared to HC subjects, patients with FM rated the stimuli as more painful (p = 0.006). In both groups, NRS ratings increased when stimulus intensity was increased (p < 0.001). No significant interaction effects were found (p = 0.099), suggesting that this effect of stimulus intensity was similar in both groups.

#### Temporal summation of pain

Fig 1B presents the NRS ratings in response to standardized electrical stimulation with 1 and 5 pulses. Statistical analysis showed that, compared to HC subjects, patients with FM rated the stimuli as more painful (p = 0.009). In both groups, NRS ratings increased when the number of pulses was increased (p = 0.001). No significant interaction effects were found (p = 0.315). As such, the effect of the number of pulses was similar in both groups, suggesting that temporal summation of pain in patients with FM was comparable to that in HC subjects.

### MEG responses to IES

The mean stimulus intensity in patients with FM was 2.89 ± 0.89 mA (NRS6). The mean stimulus intensity in HC subjects was 5.60 ± 1.95 mA (NRS6 = HC-PE). Stimulus intensities for NRS 6 were significantly lower in patients with FM as compared to HC subjects (t = 5.562, p < 0.001). Fixed stimulus intensity of 2.66 mA in HC-IE was comparable to mean stimulus intensity in patients with FM (2.89 ± 0.89 mA, NRS6).

**Fig 2:**
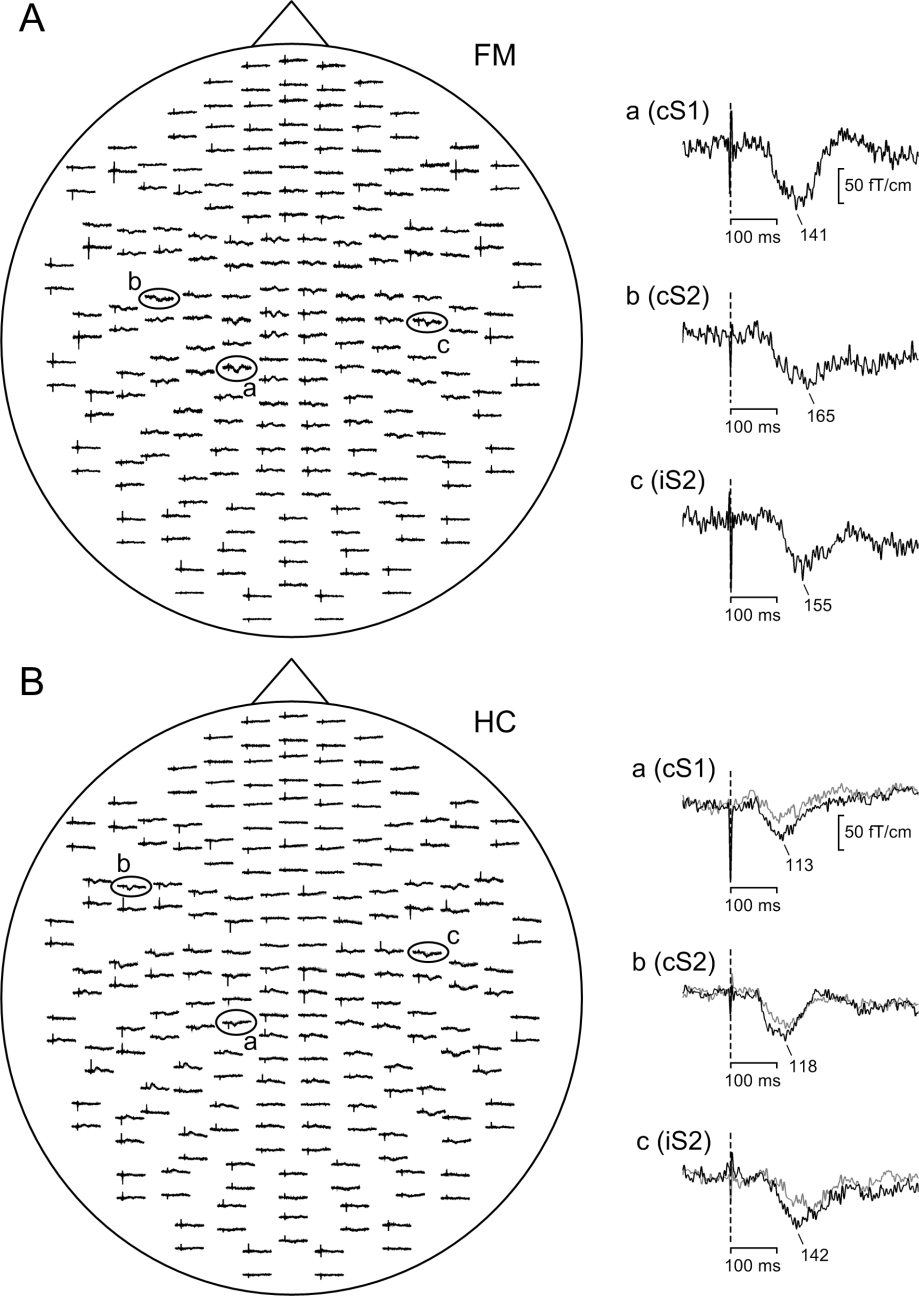
**Spatial distribution of the MEG responses in a representative FM (A) and HC subjects (B).** The head is viewed from the top. The trace of each gradiometer response pair represents the magnetic field derivation along the latitude (upper) and longitude (lower). The inserts on the right indicate the enlarged responses recorded from the contralateral S1 (a), contralateral S2 (b) and ipsilateral S2 (c) regions. The black lines represent the cortical response to the NRS 6 (FM) and perceptually-equivalent (HC) conditions. The grey line represents the cortical response to the intensity-equivalent condition (HC only). The vertical dotted lines indicate the stimulus onset.

The spatial distribution of the somatosensory evoked fields in response to noxious IES of the right hand dorsum in representative FM (A) and HC (B) subjects is presented in Fig 2. In all subjects, clear long-latency responses were observed in the contralateral parietal area (Fig 2, insert a) and the bilateral temporoparietal areas (Fig 2, insert b and c). These responses could be located in the posterior wall of the central sulcus corresponding to the location of S1, and in the upper bank of the bilateral Sylvian fissure corresponding to S2, respectively. The averaged MEG data of all subjects is in S1 Dataset (see S1 Table for description).

**Fig 3:**
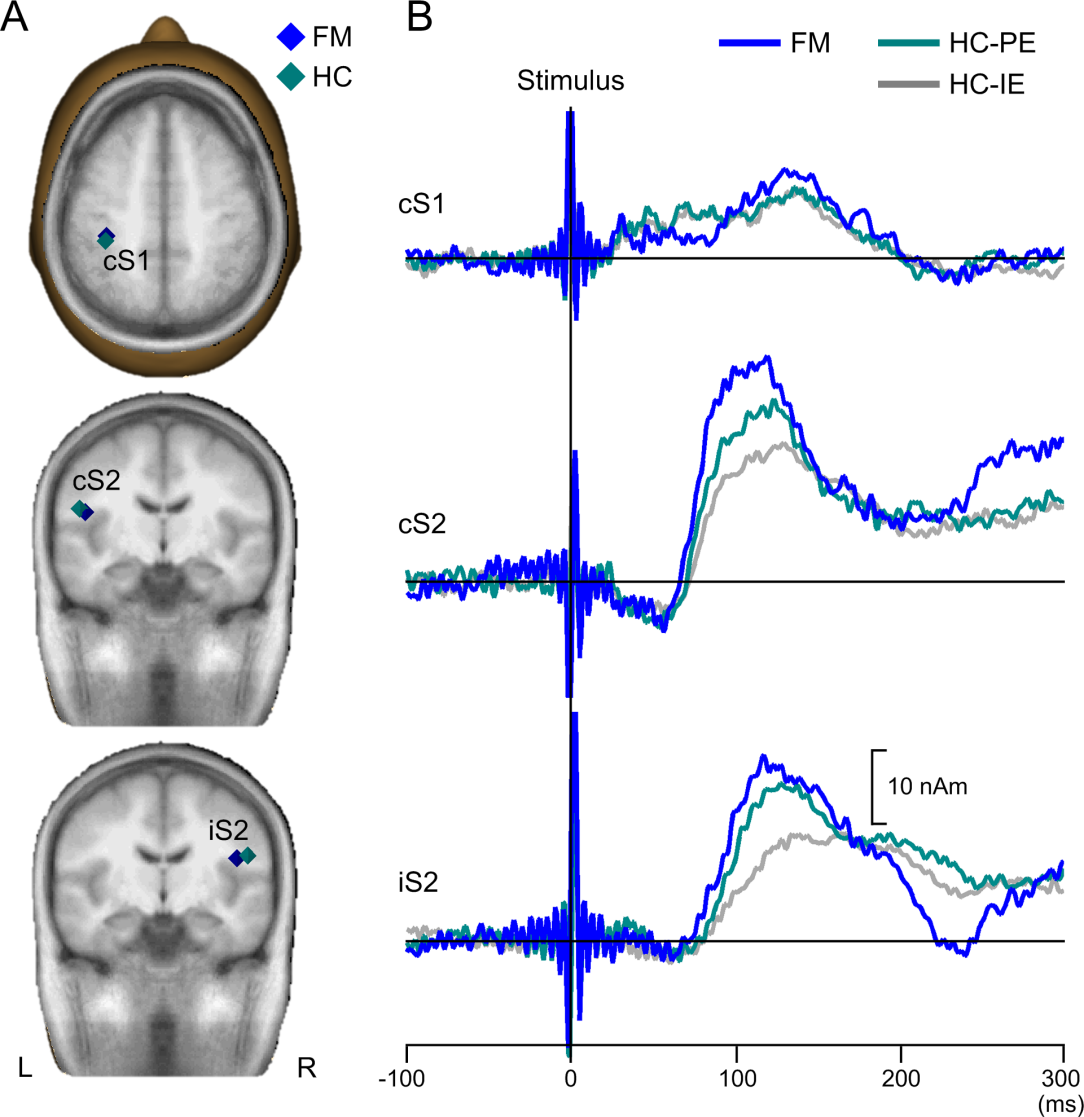
**Group mean source locations (A) and source waveforms (B).** (A) The group mean source locations for the NRS6 condition (FM) in blue and for the perceptually-equivalent condition (HC-PE) in cyan superimposed on a standard brain. (B) The group mean source waveform as a function of time. The NRS6 condition (FM) in blue, HC-PE in cyan and the intensity-equivalent condition (HC-IE) in gray. The vertical line indicates the stimulus onset.

Fig 3A shows the group mean location of the S1 and S2 dipole sources superimposed on the standard brain (Table 3). The results showed a main effect of coordinate (cS1, p < 0.001; cS2, p < 0.001; iS2, p < 0.001, respectively), whereas a main effect of group (cS1, p = 0.221; cS2, p = 0.355; iS2, p = 0.120, respectively) and a coordinate x group interaction effect (cS1, p = 0.242; cS2, p = 0.309; iS2, p = 0.103, respectively) were not observed. Fig 3B shows the grand averaged source waveforms based on the multi-dipole models as calculated for each group.

**Table 3:** Mean source locations for the NRS6 (FM) and perceptually-equivalent (HC-PE) conditions.

	FM			HC-PE		
	cS1 (n = 14)	cS2 (n = 18)	iS2 (n = 18)	cS1 (n = 15)	cS2 (n = 21)	iS2 (n = 21)
*Head coordinates*, *mm*						
*x*	-32.0 ± 8.3	-42.5 ± 7.8	41.9 ± 6.2	-31.3 ± 8.6	-47.7 ± 7.8	47.5 ± 5.9
*y*	1.7 ± 8.0	14.4 ± 8.4	15.8 ± 8.2	-1.6 ± 7.1	12.9 ± 6.4	12.9 ± 6.8
*z*	85.1 ± 8.4	58.9 ± 6.8	60.2 ± 8.4	91.6 ± 7.3	59.5 ± 8.7	61.1 ± 6.7
*Talairach coordinates*, *mm*						
*x*	-34.4 ± 6.7	-46.8 ± 7.1	43.9 ± 7.0	-35.0 ± 8.7	-50.4 ± 7.2	50.2 ± 7.8
*y*	-29.5 ± 9.5	-9.7 ± 6.4	-9.7 ± 8.0	-32.6 ± 10.6	-11.5 ± 8.2	-11.8 ± 6.2
*z*	45.5 ± 9.6	18.4 ± 7.1	21.0 ± 9.7	53.4 ± 6.0	20.6 ± 9.2	22.6 ± 8.0
GOF (%)	92.7 ± 5.8	92.4 ± 4.5	91.6 ± 5.6	90.4 ± 6.3	90.1 ± 5.7	91.3 ± 7.6

Data are expressed as the mean ± SD. Twenty HC subjects whose MRI data were available were included in deriving the source location in the Talairach coordinates. FM: fibromyalgia, HC: healthy controls, cS1: contralateral primary somatosensory cortex; cS2: contralateral secondary somatosensory cortex; iS2: ipsilateral secondary somatosensory cortex; GOF: Goodness-of-fit; n: number of subjects included in the analysis.

**Fig 4:**
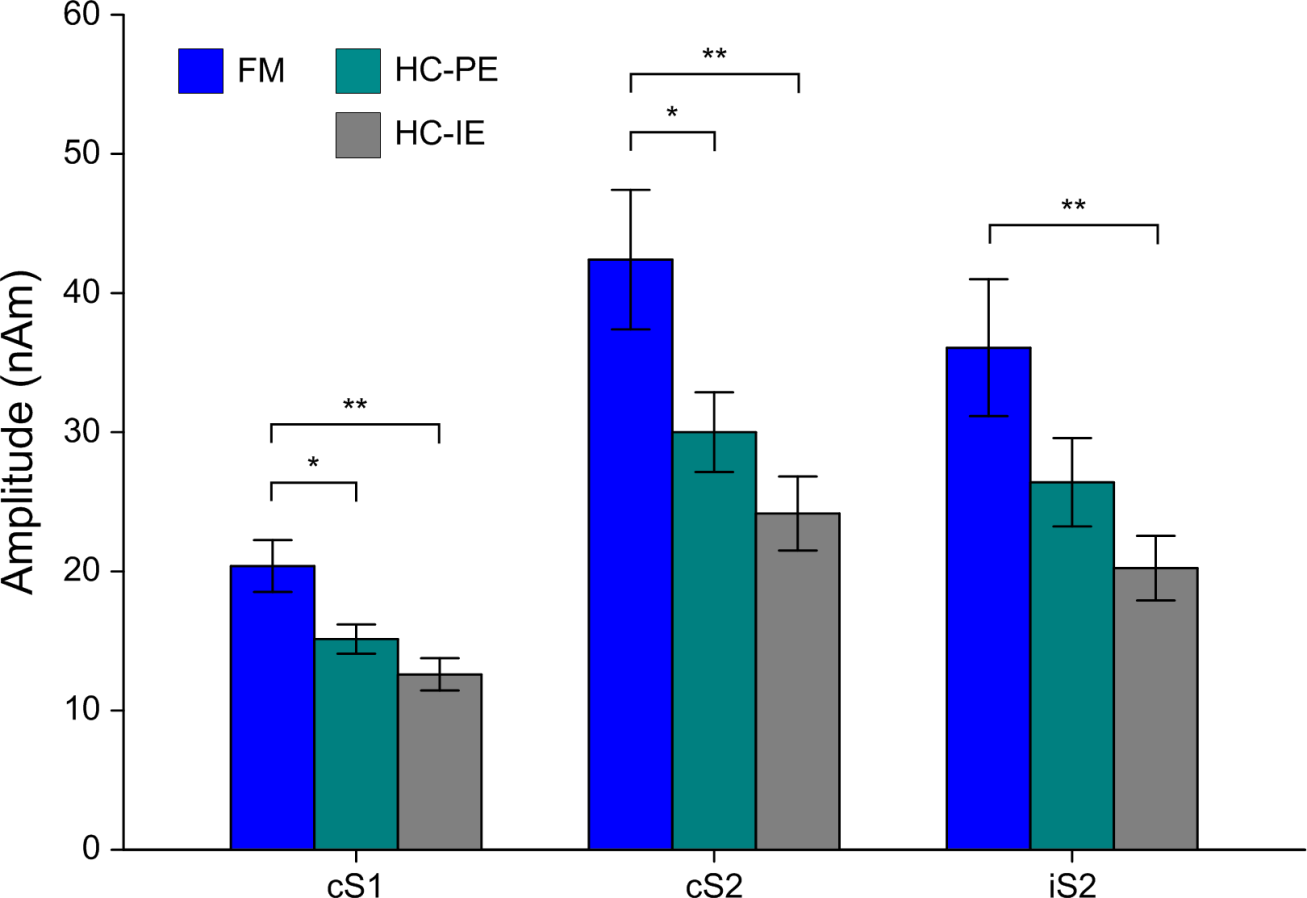
The amplitudes of the cS1, cS2 and iS2 in patients with FM and HC subjects. Repeated measures ANOVAs and independent t-tests showed that cortical responses in response to intra-epidermal electrical stimulation of the hand were higher in patients with FM as compared to both perceptually-equivalent (HC-PE) and intensity-equivalent (HC-IE) conditions in HC subjects. Data are expressed as the mean ± SEM. *p < 0.05, **p < 0.01.

Fig 4 shows the mean ± SEM amplitudes of the cS1 and bilateral S2 responses in both groups. For the amplitude of bilateral S2 responses, a significant main effect of group was found on comparing FM to both the HC-PE (F(1, 37) = 4.762, p = 0.036) and the HC-IE (F(1, 37) = 12.533, p = 0.001) conditions. The amplitudes of the S2 sources were significantly greater in patients with FM compared to HC subjects for both cS2 (HC-PE: t = 2.222, p = 0.032; HC-IE: t = 3.343, p = 0.002) and iS2 (HC-IE: t = 3.052, p = 0.004). The amplitude of cS1 responses was significantly greater in patients with FM compared to HC subjects for both the HC-PE (t = 2.496, p = 0.019) and HC-IE (t = 3.599, p = 0.001) conditions. Peak latencies of the S1 and S2 sources (Table 4) were not different between groups.

**Table 4:** Peak latencies and peak amplitudes of the S1 and S2 sources.

	Latency (ms)			Amplitude (nAm)		
	cS1	cS2	iS2	cS1	cS2	iS2
FM	137.7 ± 28.7	129.5 ± 27.5	148.1 ± 28.5	20.4 ± 7.0	42.4 ± 21.3	36.1 ± 20.9
HC-PE	132.8 ± 26.5	120.8 ± 13.6	137.5 ± 23.4	15.1 ± 4.1	30.0 ± 13.1	26.4 ± 14.6
HC-IE	135.6 ± 29.2	125.0 ± 17.1	146.0 ± 22.1	12.6 ± 4.5	24.2 ± 12.2	20.2 ± 10.6

Data are expressed as mean ± SD. FM: fibromyalgia; HC-PE: perceptually-equivalent condition in healthy controls; HC-IE: intensity-equivalent condition in healthy controls; cS1: contralateral primary somatosensory cortex; cS2: contralateral secondary somatosensory cortex; iS2: ipsilateral secondary somatosensory cortex.

**Fig 5:**
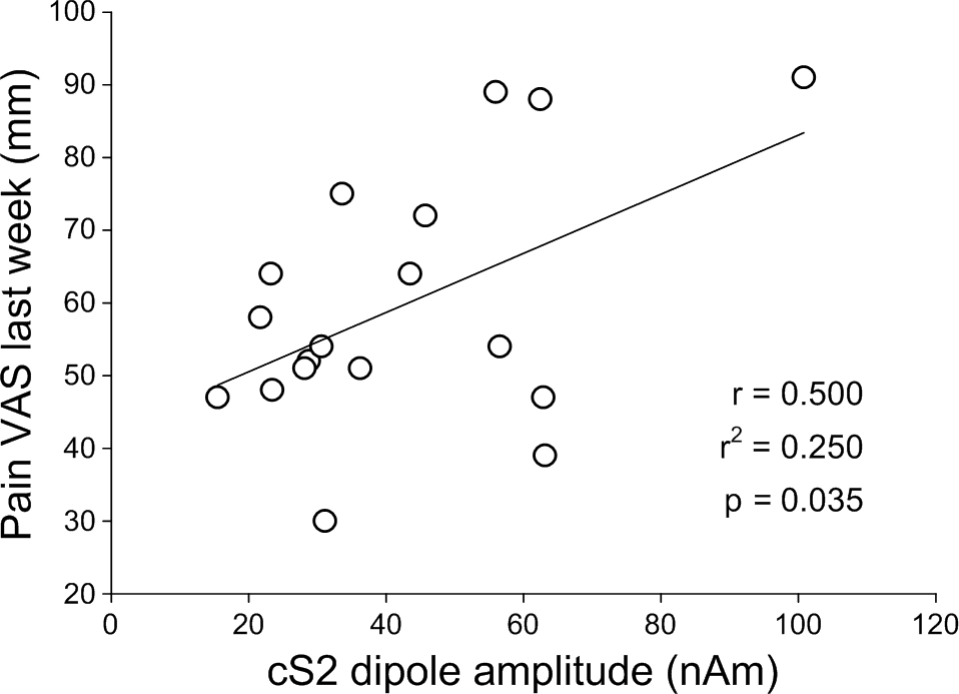
Relationship between the dipole amplitude in the cS2 and clinical pain intensity (VAS, mm).

The amplitude of the cS2 response was positively correlated to the clinical pain intensity over the last week (r = 0.500, p = 0.035) (Fig 5). In an exploratory analysis, no significant correlations were found between the amplitude of the cortical response and the sensory and affective pain scores (SF-MPQ).

## Discussion

The present study aimed to investigate the central augmentation of pain processing in patients with FM using MEG and IES. Dipole amplitudes in the cS1, cS2 and iS2, were found to be increased in patients with FM as compared to HC subjects, and the amplitude of the cS2 response in patients with FM was positively correlated to the clinical pain intensity reported over the last week. In addition, QST findings showed that patients with FM were more sensitive to painful IES as well as to mechanical stimulation, regardless of whether the stimulation site was the hand or the trapezius muscle. Interestingly, the slope of the stimulus-response relationship as well as temporal summation of pain in response to IES was not different between groups, suggesting that the observed pain augmentation in response to IES in patients with FM could be due to sensitization or disinhibition at the cortical level.

### Quantitative sensory testing

Our findings showing no differences in tactile as well as electrical detection threshold between the groups suggest that tactile sensitivity in patients with FM is generally normal. This result is in line with several other studies that showed no differences between FM patients and HC subjects in terms of tactile perception threshold [65] and electrical [66, 67] and mechanical detection threshold [4]. In contrast to the findings of tactile sensitivity, FM patients showed increased pain sensitivity to IES as well as to mechanical stimulation, regardless of whether they were stimulated at a tender point (trapezius) or a neutral site (hand), indicating the absence of specific changes at tender points. However, the stimulus-response curve slope was similar in patients with FM and HC subjects, suggesting that the increased pain sensitivity in patients with FM was due to a higher offset in stimulus intensity perception, rather than being intensity dependent. This is consistent with previous FM studies demonstrating a parallel leftward shift in the stimulus-response curve without a change in slope in response to painful heat and pressure stimuli [65, 68]. These results confirm and extend previous work reporting reduced pain thresholds and increased pain ratings in patients with FM in response to a variety of different stimuli at different body sites [3–7, 22, 69, 70], and point towards central sensitization or disinhibition. This finding is in contrast with several recent studies implicating a role for peripheral abnormalities in the maintenance of FM [71, 72]. Although peripheral mechanisms cannot be ruled out, our results suggest that peripheral sensitization is unlikely to explain the observed pain augmentation in response to IES in our patients with FM.

In addition to sensory thresholds and ratings, we assessed temporal summation of pain in response to IES, which could be considered as the electrical equivalent of a noxious pinprick stimulus, as used in studies by the German Research Network on Neuropathic Pain [58]. Temporal summation of pain refers to the increase in perception of pain with repetitive noxious stimuli (≥0.3 Hz) that is thought to be related to a frequency-dependent increase in the excitability of spinal cord neurons, commonly known as wind-up [10, 73]. In the present study, patients with FM reported higher ratings in response to single and repeated IES, but temporal summation of pain was not significantly different from that observed in HC subjects. This is consistent with several other studies which showed that patients with FM were not significantly different from HC subjects when testing temporal summation of pain using pinprick [4, 74, 75] or thermal stimuli [30], or when testing reflex responses to nociceptive electrical stimulation [29]. In contrast, several other studies have reported increased temporal summation of pain in response to thermal stimuli [10, 11], and reduced reflex responses to nociceptive electrical stimulation in patients with FM [6, 76]. These mixed results are likely due to clinical heterogeneity of the FM symptoms and methodological differences in stimulation. It can be argued that the use of pain thresholds for the assessment of temporal summation can be problematic due to the low pain thresholds commonly observed in patients with FM [30]. Therefore, more carefully designed studies are needed to clarify the presence or absence of abnormal temporal summation of pain in patients with FM.

What is interesting about the findings in our study as compared to those in other studies is that we used the same type of stimulus (IES) for the assessment of temporal summation of pain, sensory thresholds and ratings, and cortical responses. The lack of differences between patients with FM and HC subjects with respect to temporal summation of pain in response to IES as observed in this study raises questions about the role of spinal sensitization in explaining the observed pain augmentation in response to IES in our patients with FM.

### MEG responses to IES

After noxious stimulation of the skin, several cortical sources may be identified including the S1, S2, insular, and anterior cingulate cortex [77]. These cortical sources are considered to be part of the so-called ‘pain matrix’, a network of brain areas implicated in the processing of pain [78]. To the best of our knowledge, this study is the first research to combine MEG and IES in the assessment of patients with FM and several sources could be identified, including the bilateral S2 and cS1. Increased activity in the cS2 and cS1 was observed in patients with FM when compared to both perceptually and intensity controlled activation in HC subjects. This is consistent with a previous MEG study that used subjectively matched painful pressure stimuli in patients with FM and HC subjects [17]. Lower stimulus intensities were required to evoke pain in patients with FM. However, these lower stimulation intensities still resulted in increased evoked fields in the somatosensory, temporal and parietal areas at short latencies, and in prefrontal areas at both short and long latencies in patients with FM as compared to HC subjects [17]. Importantly, in our study, significantly increased activity in the ipsilateral S2 was observed, an area that has recently been implicated in mediating clinical pain in patients with FM [18]. Since the S2 has been shown to play a role in higher-order functions, such as attention [79] and integration of nociceptive and non-nociceptive sensory input [80], further studies are needed to clarify the role of augmented S2 response in clinical characteristics of FM.

Augmented cortical responses to nociceptive stimuli could be due to facilitation or disinhibition of nociceptive input at multiple levels in the cortical pain matrix. For example, it has been suggested that prolonged nociceptive input could lead to central sensitization and maladaptive neuroplasticity within the somatosensory and motor systems [81]. In our study by utilizing paired-pulse median nerve stimulation and MEG, we found that intracortical inhibition in the S1 is compromised in FM patients [23]. In addition, a growing body of evidence has suggested that deficits in endogenous inhibitory systems could play an essential role in FM [51, 82–84]. A recent functional MRI study demonstrated that activity of the rostral anterior cingulate cortex, known to play a crucial role in descending pain inhibition, was attenuated in patients with FM upon painful pressure stimulation [82]. Moreover, patients with FM showed reduced functional connectivity between the rostral anterior cingulate cortex and other regions involved in the descending pain inhibitory network including the brainstem, rostral ventromedial medulla, amygdala, and hippocampus [83]. Lastly, several previous studies assessing pain augmentation in patients with FM have also reported decreased thalamic activation in response to both painful [13] and non-painful [12] stimuli. As such, the observed pain augmentation in the present study could have been due to altered filtering of nociceptive input in the thalamus.

### Methodological considerations

In this study we used tools that have not been previously used (IES) or have been infrequently (MEG) applied in the study of FM. The advantage of MEG is that cortical responses can be measured in the time and space domain in great detail. However, only a subset of brain regions involved in pain processing can be reliably detected using this method. To obtain information about concomitant activation in other brain regions, other imaging methods as well as more complex analysis procedures [17, 85] may complement the present results.

A particular strong point of our study was the assessment of cortical responses to IES in the context of behavioral responses to the same type of stimulus. Still, in order to do so, different levels of stimulation were used. Previous studies often used IES at or just above pain threshold levels (<2.5 mA) [36, 37, 85], to ensure Aδ-fiber specific stimulation [37]. In some of our subjects (including both patients and HC subjects), higher stimulus intensities were occasionally needed, e.g. to assess the EPTT or to attain a perceptual pain rating of 6 on the NRS. This means that the data cannot be contributed to the Aδ-fiber function alone. If IES activates Aδ- and Aβ-fibers simultaneously, Aβ stimulation may have interfered with perception of pain elicited by noxious stimuli. A study by Inui et al. found that cortical responses to noxious IES can be inhibited by applying simultaneously innocuous transcutaneous electrical stimulation [86]. This effect may be more pronounced in case of HC-PE, where HC subjects required higher stimulus intensities to induce moderate pain. However, systematic studies examining to what extent the noxious evoked brain response was reduced by Aβ co-activation according to increasing intensity of IES have not yet been performed. In addition, the subjective sensation of the stimulus as well as the cortical response latencies comparable to those observed in studies using lower stimulus intensities suggested a primary activation of Aδ-fibers. Markedly increased amplitudes of cS1, cS2 and iS2 responses in FM compared to HC-IE condition further support our interpretation of pain augmentation in response to IES in FM. Taken together, although our results cannot exclude the possibility that a higher intensity stimulus activates other kinds of fibers, it is unlikely that this potential limitation affected the between-group differences in response to IES. In the light of recent studies reporting C-fiber abnormalities in patients with FM [71, 72], it could additionally be interesting to repeat the present study using a recently developed modified version of the IES electrode that selectively stimulates C-fibers [87].

Lastly, although the sample size in the present study was larger than that in previous clinical MEG studies, the sample size was still relatively small considering the heterogeneous presentation of FM. In addition, the unknown effects of medication (or rather the medication stoppage and limited washout period) limit the generalizability of the present results.

## Conclusion

The present study investigated the central augmentation of pain processing in patients with FM using MEG and IES. Increased responses were found in the cS1, cS2 and iS2 in patients with FM, and the amplitude of the cS2 response was found to be positively correlated with clinical FM pain intensity. These results corresponded with generalized hypersensitivity to both IES and mechanical stimuli as assessed using QST. Temporal summation of pain in response to IES was not different between patients with FM and HC subjects. Together, these results suggest that the observed pain augmentation in response to IES in patients with FM occurred relatively independent of peripheral and spinal sensitization to IES. The observed pain augmentation in response to IES in patients with FM could be due to sensitization or disinhibition of the cortical somatosensory system. The combination of MEG and IES could therefore be particularly useful to explore the role of bilateral somatosensory cortices in FM pathophysiology.

## Supporting Information

S1 Dataset Averaged MEG data. (ZIP)

S1 Table Description of variables in the MAT-file. (DOCX)
