# Supplementary material for: Augmented Pain Processing in Primary and Secondary Somatosensory Cortex in Fibromyalgia: A Magnetoencephalography Study Using Intra-Epidermal Electrical Stimulation
Source: PLoS One. 2016 Mar 18;11(3):e0151776. doi: 10.1371/journal.pone.0151776 (PMC4798786; doi:10.1371/journal.pone.0151776)
Supplement: S1 Table — (DOCX) [file pone.0151776.s002.docx]

**S1 Table. Description of variables in the MAT-file.**

| Name | Value | Description |
| --- | --- | --- |
| B | n x m double | The data of one channel in each row, a single time sample in each column.  ***Units:*** *SI units. T/m for gradiometers, T for magnetometers.* |
| COILTYPE | n x 1 double | Coil type (3012, gradiometer; 3024, magnetometer) |
| NAME | n x 1 cell array | The channel names |
| sfreq | double | Sampling frequency  ***Units:*** *Hz* |
| t0 | double | Start time of the epoch. The analysis period of 0.5 s included a  pre-stimulus baseline of 0.1 s.  ***Units:*** *s* |
